# Influenza viruses that require 10 genomic segments as antiviral therapeutics

**DOI:** 10.1371/journal.ppat.1008098

**Published:** 2019-11-15

**Authors:** Alfred T. Harding, Griffin D. Haas, Benjamin S. Chambers, Nicholas S. Heaton

**Affiliations:** Department of Molecular Genetics and Microbiology, Duke University School of Medicine, Durham, NC, United States of America; University of Pennsylvania, UNITED STATES

## Abstract

Influenza A viruses (IAVs) encode their genome across eight, negative sense RNA segments. During viral assembly, the failure to package all eight segments, or packaging a mutated segment, renders the resulting virion incompletely infectious. It is known that the accumulation of these defective particles can limit viral disease by interfering with the spread of fully infectious particles. In order to harness this phenomenon therapeutically, we defined which viral packaging signals were amenable to duplication and developed a viral genetic platform which produced replication competent IAVs that require up to two additional artificial genome segments for full infectivity. The modified and artificial genome segments propagated by this approach are capable of acting as “decoy” segments that, when packaged by coinfecting wild-type viruses, lead to the production of non-infectious viral particles. Although IAVs which require 10 genomic segments for full infectivity are able to replicate themselves and spread *in vivo*, their genomic modifications render them avirulent in mice. Administration of these viruses, both prophylactically and therapeutically, was able to rescue animals from a lethal influenza virus challenge. Together, our results show that replicating IAVs designed to propagate and spread defective genomic segments represent a potent anti-influenza biological therapy that can target the conserved process of particle assembly to limit viral disease.

## Author summary

Influenza infections are best prevented via prophylactic vaccination. Vaccination, however, is incompletely efficacious, necessitating the use of anti-influenza therapeutics. To date, several different classes of anti-influenza therapeutics have been developed and used in order to combat these infections. Unfortunately, the incidence of influenza resistance to many of these therapeutics has begun to rise, necessitating the development of new strategies. One such strategy is to mimic the activity of naturally occurring viral particles that harbor defective genomes. These defective interfering particles have the ability to interfere with productive viral assembly, preventing the spread of influenza viruses across the respiratory tract. Furthermore, given the manner in which they target influenza segment packaging, a conserved feature of all influenza A viruses, resistance to this therapeutic strategy is unlikely. Here, we report the development of a genetic platform that allows the production of replicating influenza viruses which require 10 genomic segments to be fully infectious. These viruses are capable of amplifying themselves in isolation, but coinfection with a wild-type virus leads to segment exchange and compromises the spread of both viruses. This interference, while mechanistically distinct from naturally occurring defective particles, was able to target the same viral process and rescue animals exposed to an otherwise lethal viral infection. This viral-based approach may represent a cost effective and scalable method to generate effective anti-influenza therapeutics when vaccines or antiviral drugs become ineffective due to the acquisition of viral resistance mutations.

## Introduction

Influenza virus infections represent a substantial global burden on human health. Each year, it is estimated that influenza viruses cause up to 5 million severe infections globally, resulting in up to 645,000 mortalities [1, 2]. In 2018, patient care and productivity loss due to influenza infection cost an estimated $11.2 billion in the U.S. alone [3]. Influenza A virus (IAV), is the major contributor to total influenza disease, and the primary measure used to control IAV spread is prophylactic immunization. However, due to rapid viral antigenic drift, vaccination can have limited efficacy and therefore therapeutic small molecules are also used for treating influenza disease. Amantadines, which inhibit the influenza M2 ion channel were the first IAV therapeutics developed, and were approved for clinical use in 1966 [4]. Point mutations in the channel itself, however, can lead to resistance to these inhibitors, and high levels of resistance are now widespread in H1, H3, H5, H7, H9, and H17 subtype influenza A viruses [5–7]. Neuraminidase inhibitors, such as oseltamivir, are now the most commonly used IAV therapeutic [8]. However, this class of inhibitors suffers from viral resistance as well [9–11]. Recently, an mRNA cap-snatching inhibitor, Baloxavir, was FDA-approved [12, 13], and the rate at which viral resistance may be acquired is currently unknown. Due to the limited timeframes after infection in which these therapeutics are effective, as well as the threat of emerging viral resistance, additional antiviral therapeutics are currently in various stages of development [12]. One promising alternative therapeutic approach, that theoretically would be difficult for IAVs to develop resistance to, has been the utilization of defective viral particles that disrupt viral replication and packaging [14].

Defective viral particles are produced by, and important for, a number of different RNA viruses [15–17]. For influenza viruses, these particles represent replication-incompetent virions that frequently harbor one or more viral genomic segments with a significant deletion of the open reading frame (ORF) of that segment [18]. Internal deletions can occur spontaneously during the replication stage of the viral lifecycle and lead to the formation of a genomic segment that fails to include most of the ORF but retains the 5’ and 3’ packaging signals necessary for gene segment incorporation [19, 20]. If this partially deleted segment is packaged into nascent virions, these particles are capable of infecting a host cell, but are then unable to produce viable progeny due to the absence of the protein normally encoded by the defective genomic segment [21]. While these defective interfering particles (DIPs) are replication incompetent, they can be successfully propagated during coinfection with another defective or a fully infectious viral particle. Although it was believed that such coinfections are relatively uncommon, recent work has shown that coinfection may actually be an important contributor to productive virus replication [22]. Furthermore, when DIP coinfection occurs, the defective segment(s) of the interfering particle are thought to be replicated more quickly than their wild-type counterparts due to their significantly smaller size [14, 23–25].

The disruptive effect of DIPs has garnered attention as a potential influenza antiviral treatment [26–28]. Studies have shown that laboratory-produced DIPs can be used prophylactically and therapeutically to protect mice and ferrets from IAV infection [29, 30]. This DI system has also been shown to be effective *in vitro* in human respiratory tract cell lines [31]. Despite these advances, options for generating DIPs have been limited. Initially, DIPs were synthesized via high multiplicity passaging, which not only generates diverse DI populations with varying efficacy, but also contains wild-type IAVs that must be inactivated by UV irradiation [32, 33]. Reverse genetic cloning has offered a means through which to generate populations of specific DIP genotypes, however this method requires the use of helper viruses for the proliferation of the DIPs, which still necessitates subsequent UV inactivation.

We were interested in generating IAV mutants that therapeutically mimic the inhibitory activity of DIPs, but using a fundamentally different molecular strategy. We hypothesized that a live-attenuated virus population harboring and amplifying synthetic artificial genome segments could interfere with wild-type viral spread via the cross-packaging of the artificial genomic segments during a coinfection. In this report, we describe the genomic design of an IAV strain that requires the presence of 10 genomic segments (10S virus) to be fully infectious that accomplishes this goal. Administration of 10S viruses either prophylactically or therapeutically rescued animals from an otherwise lethal viral infection. Thus, reprogramming an IAV viral genome to interfere with normal viral spread is a viable approach and one that may be less susceptible to the development of viral resistance, as the target is the generally conserved process of viral genome assembly.

## Results

### Evaluation of viral genetic manipulations capable of generating IAVs that require 9 genomic segments

We were interested in generating influenza viruses that could be genetically programmed to harbor artificial genomic segments that would interfere with the genome assembly of WT-IAV strains. It was previously reported that the NA packaging signals could be duplicated and utilized to propagate a ninth genomic segment [34]. While this approach was initially used to encode additional antigens as a vaccine platform, we theorized that this and similar approaches could be utilized to generate viruses harboring artificial, interfering segments. We therefore tested the ability to duplicate various packaging signals and generate viruses that required 9 segments (9S viruses) to be fully infectious in the A/Puerto Rico/8/1934 (PR8) genetic background. We tested the incorporation of additional segments via the duplication of NA, NP, HA, and PA packaging signals in different combinations (Table 1, S1 Fig). In all cases, the “9^th^” segment was designed encoding super-folder GFP (sfGFP) or mCherry and harboring unique packaging signals so that it would always be packaged and failure to package the duplicated packaging signal segment would lead to the loss of an essential viral protein. Surprisingly, very few segment duplications were amenable to this approach. As previously reported, duplication of the NA packaging signal is tolerated, but of all of the other segments tested, only duplication of the PA packaging signal was tolerated (Table 1).

**Table 1:** Design strategies for IAV genomes that propagate 9 genomic segments. A description of the manipulated packaging signals, encoded proteins, and success of rescuing each 9 segmented IAV strategy.

	Segment Design	Protein	Successful Rescue		Segment Design	Protein	Successful Rescue
	**9S PB1-mCherry-PB1 / NA-PB1-NA Design**				**9S PB2-sfGFP-PB2 / PA-PB2-PA Design**		
1	WT PB2	PB2	**Yes** (Gao *et al*.) PMID: 20519387	1	**PA-PB2-PA**	PB2	**Yes**
2	**NA-PB1-NA**	PB1		2	WT PB1	PB1	
3	WT PA	PA		3	WT PA	PA	
4	WT HA	HA		4	WT HA	HA	
5	WT NP	NP		5	WT NP	NP	
6	WT NA	NA		6	WT NA	NA	
7	WT M	M1, M2		7	WT M	M1, M2	
8	WT NS	NS1, NEP		8	WT NS	NS1, NEP	
9	**PB1-mCherry-PB1**	mCherry		9	**PB2-sfGFP-PB2**	sfGFP	
	**9S PB2-sfGFP-PB2 / NP-PB2-NP Design**				**9S M-zsGreen|M2-M / HA-M1-HA Design**		
1	**NP-PB2-NP**	PB2	No	1	WT PB2	PB2	No
2	WT PB1	PB1		2	WT PB1	PB1	
3	WT PA	PA		3	WT PA	PA	
4	WT HA	HA		4	WT HA	HA	
5	WT NP	NP		5	WT NP	NP	
6	WT NA	NA		6	WT NA	NA	
7	WT M	M1, M2		7	**HA-M1-HA**	M1, M2	
8	WT NS	NS1, NEP		8	WT NS	NS1, NEP	
9	**PB2-sfGFP-PB2**	sfGFP		9	**M-zsGreen | M2-M**	mCherry	
	**9S HA-sfGFP-HA / NS-HA-NS Design**				**9S NS-mCherry|NEP-NS / NA-NS1-NA Design**		
1	WT PB2	PB2	No	1	WT PB2	PB2	**Yes** (Unstable)
2	WT PB1	PB1		2	WT PB1	PB1	
3	WT PA	PA		3	WT PA	PA	
4	**NS-HA-NS**	HA		4	WT HA	HA	
5	WT NP	NP		5	WT NP	NP	
6	WT NA	NA		6	WT NA	NA	
7	WT M	M1, M2		7	WT M	M1, M2	
8	WT NS	NS1, NEP		8	**NA-NS1-NA**	NS1, NEP	
9	**HA-sfGFP-HA**	sfGFP		9	**NS-mCherry | NEP-NS**	mCherry	

We also tested the potential of using splice sites in the 7th and 8th segments of IAV to generate 9S viruses expressing either M1 or NS1 in the ninth segment and a fluorescent protein with M2 or NEP in segment 7 or 8, respectively (Table 1, S1 Fig). Duplicating HA packaging signals and encoding M1 as an artificial segment was unsuccessful. However, duplicating the NA packaging signals and encoding NS1 successfully yielded 9S virions. The “NS” segment encoding mCherry and NEP however, immediately lost mCherry signal upon viral rescue, indicating that this approach is not useful for the stable incorporation of protein or nucleic acid. From these experiments we conclude that the duplication of packaging signals is not a generalizable approach for all segments, but in some specific cases, such as with NA and PA packaging signals, this approach can be utilized to force viral propagation of a 9^th^ genomic segment.

### Characterization of 9S viruses and their therapeutic efficacy

**Fig 1:**
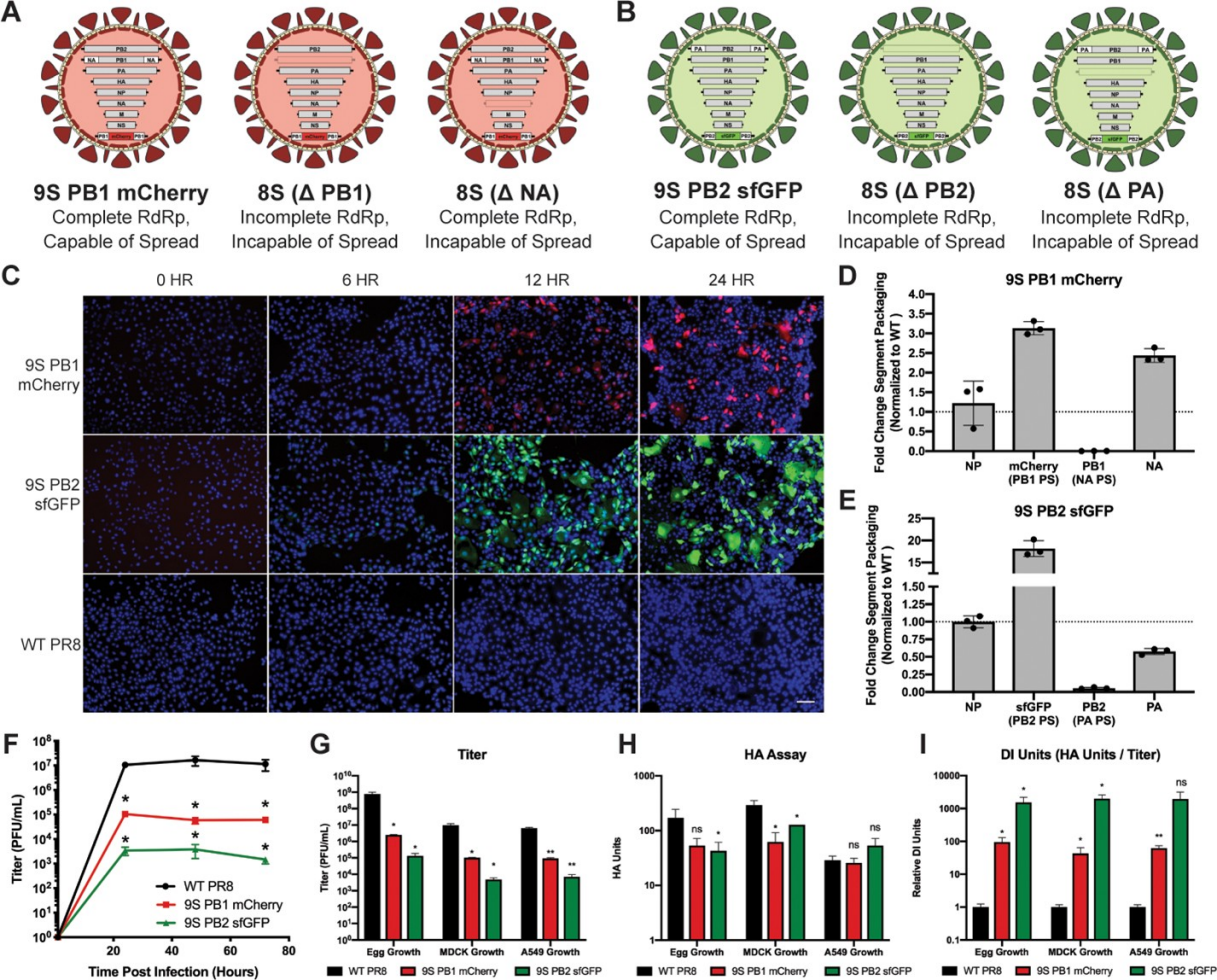
Viruses that require 9 genomic segments for infectivity generate more defective particles than the parental strain. (A and B) Potential genomic configurations of the 9S PB1 mCherry virus (A) and the 9S PB2 sfGFP virus (B) assuming packaging of at least 8 segments. (C) Fluorescent microscopy images of 9S PB1 mCherry, 9S PB2 sfGFP, or WT PR8 virus-infected MDCK cells at 6, 12, and 24 hours post-infection at an MOI of 0.1; nuclei were stained blue using DAPI staining and the scale bar represents 100 micrometers. (D and E) qRT-PCR of specific vRNA segments of the 9S PB1 mCherry virus (D) and the 9S sfGFP virus (E) after growth in eggs normalized to wild-type PR8 segment levels. Artificial segments were normalized based on the packaging signals of that segment (i.e. the PB1 mCherry segment was normalized to wild-type PB1). (F) Multicycle-growth curve of 9S PB1 mCherry, 9S PB2 sfGFP, and WT PR8 viruses in MDCK cells (MOI = 0.05). (G) Endpoint titer 72 hours post-infection in 10-day old embryonated chicken eggs, Madin-Darby canine kidney cells and A549 human lung-epithelial cells of the indicated viruses (MOI = 0.05). (H) HA assays 72 hours post-infection in 10-day old embryonated chicken eggs, Madin-Darby canine kidney cells and A549 human lung-epithelial cells of the indicated. (I) The “DI Units” of the 9-segmented fluorescent viruses as compared to that of WT PR8 virus, calculated by dividing respective normalized HA units by normalized endpoint titer. For all graphs, * represents a p-value of ≤ .05 and ** represents a p-value of ≤ .001.

We next began *in vitro* characterization of the 9S PB1 mCherry virus, with duplicated NA packaging signals, and the 9S PB2 sfGFP virus, with duplicated PA packaging signals. We hypothesized that our genomic design could potentially lead to three major populations of virions: One that packaged all 9 segments, or two different eight-segmented viruses which failed to package one of the segments with duplicated packaging signals (Fig 1A & 1B). After infection of MDCK cells with these viruses, we observed the expected fluorescence (Fig 1C), and co-positivity for the viral hemagglutinin (HA) protein was observed at approximately the same rate as an eight-segmented fluorescent reporter virus (S2 Fig). These results indicate that, as designed, the artificial genomic segments (which have unique packaging signals) are present when viral replication occurs. These data however, did not report on the relative packaging of all of the manipulated viral segments. We therefore designed segment specific qRT-PCR assays and observed that our 9S viral particles package variable levels of different genomic segments (Fig 1D and 1E). Consistent with our design and microscopy data, the fluorescent genomic segments (PB1 mCherry & PB2 sfGFP) were packaged very efficiently, but the modified segments encoding viral proteins with duplicated packaging signals exhibited drastically reduced packaging relative to their matched packaging signal counterparts (e.g. the segment with NA PS harboring PB1 vs the WT NA segment).

We next began *in vitro* characterization of the 9S viruses, and multicycle growth on MDCK cells revealed a significant reduction in viral titer (Fig 1F). Since this reduction was likely due to progeny virions failing to package one of the required segments, we grew the viruses under multiple conditions and measured both infectious particles via plaque assay and also performed a hemagglutinin (HA) assay, which measures both infectious and noninfectious particles (Fig 1G and 1H). Regardless of growth conditions, we again observed a major reduction in viral titer, while the magnitude of the observed defect in the HA assay was much smaller. To represent this difference, we calculated the “relative DI units” of our 9S viruses, relative to WT PR8 virus set at an arbitrary value of 1, by dividing HA units by the endpoint titer (Fig 1I). From this analysis, we conclude that the 9S PB1 mCherry virus produced ~100x times more defective viral progeny and the 9S PB2 sfGFP virus produced ~1000 times more non-viable progeny than did WT PR8 regardless of where the viruses were propagated (Fig 1I). Finally, in order to test if these genomic modifications were only applicable to the PR8 viral background, we cloned a 9S PB2 sfGFP virus in the unrelated H3N2 A/Wyoming/03/03 background. We were able to successfully rescue this virus (S3 Fig), demonstrating that these genomic manipulations are not exclusively tolerated in laboratory-adapted H1N1 IAV strains.

**Fig 2:**
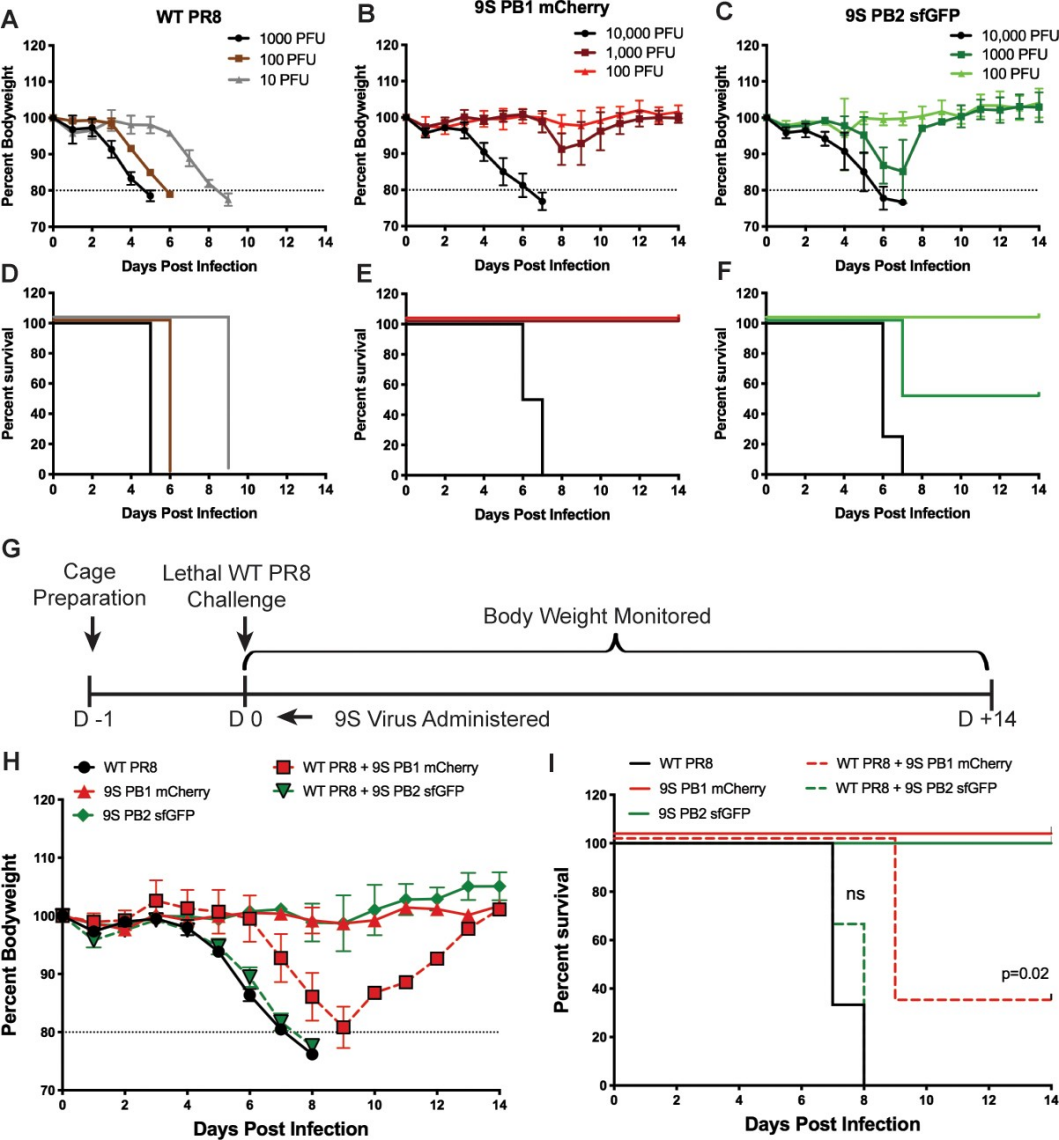
Viruses that require 9 genomic segments are highly attenuated and can interfere with lethal viral challenge. (A–C) Weight loss curves from infections with the indicated doses of WT PR8 virus (A), the 9S PB1 mCherry virus (B), or the 9S PB2 sfGFP virus (C). (D-F) Survival curves from infections with the indicated doses of WT PR8 virus (D), the 9S PB1 mCherry virus (E), or the 9S PB2 sfGFP virus (F). (G) Experimental design of the coinfection challenges in C57BL/6J mice. (H) Weight-loss curves from infecting mice with a lethal dose of WT PR8 (20 PFU), a sublethal dose of the 9S PB1 mCherry virus (500 PFU), a sublethal dose of the 9S PB2 sfGFP virus (500 PFU) a combination of WT PR8 and the 9S PB1 mCherry virus, or a combination of WT PR8 and the 9S PB2 sfGFP virus. (I) Survival curves from challenges described in H.

We next wanted to assess how requiring a 9^th^ genomic segment for full infectivity would affect virulence in C57BL/6 mice, as well as determine if 9S fluorescent viruses could interfere with influenza disease. We therefore administered increasing doses of the virus and while the parental PR8 virus induced lethal disease at all doses tested, the two 9S viruses were significantly attenuated (Fig 2A–2F). The 9S PB1 mCherry virus required 10^4^ PFU for lethal disease (Fig 2B and 2E) while the 9S PB2 sfGFP was slightly more virulent, and some mortality was observed at the 10^3^ PFU dose (Fig 2C and 2F). Based on this attenuation, and the presumed large amount of DI particles being produced *in vivo*, we next assessed the capability of each 9-segmented fluorescent virus to interfere with a lethal PR8 infection. We coinfected animals with 20 PFU of WT virus in combination with 500 PFU of either the 9S PB1 mCherry or the 9S PB2 sfGFP virus and monitored morbidity and mortality for 14 days (Fig 2G). Non-treated control animals rapidly lost body weight and reached humane endpoints as expected (Fig 2H and 2I). Co-administration of the 9S PB2 sfGFP virus did not significantly reduce weight loss or increase survival, however the 9S PB1 mCherry virus induced a measurable protective effect in both the kinetics of weight loss and increased animal survival (Fig 2H and 2I).

### The artificial viral segment size modestly affects 9S virus therapeutic efficacy

**Fig 3:**
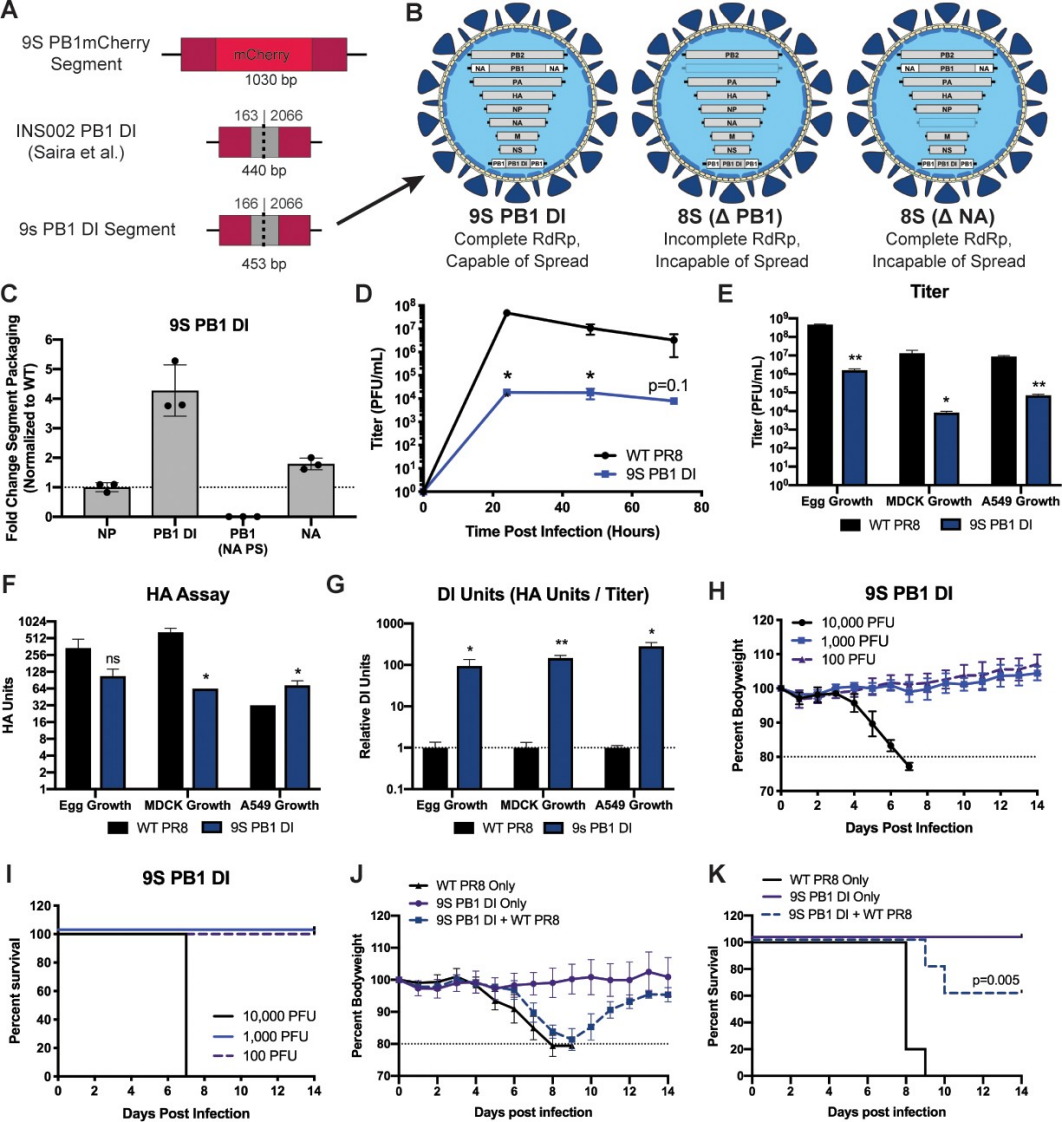
Influenza viruses can harbor and propagate an artificial, defective interfering-like genomic segment. (A) A schematic comparing the 9S PB1-mCherry-PB1 segment and the INS002 PB1 DI segment, which acted as a basis for the design of the 9S PB1 DI segment. (B) Potential genomic configurations of the 9S PB1 DI virus assuming packaging of at least 8 genomic segments. (C) qRT-PCR of specific vRNA segments of the 9S PB1 DI virus after growth in eggs normalized to wild-type PR8 segment levels. Segments were normalized based on the intact packaging signals of that segment (i.e. the PB1 DI segment was normalized to wild-type PB1) (D) Multicycle growth curve of the 9S PB1 DI virus on MDCK-cells infected at an MOI of 0.05. (E) Endpoint titer 72 hours post-infection in embryonated chicken eggs, Madin-Darby canine kidney cells (MOI = 0.05), and A549 human lung-epithelial cells (MOI = 0.05) of the 9S PB1 DI and WT PR8 virus. (F) HA assay 72 hours post-infection in 10-day old embryonated chicken eggs, Madin-Darby canine kidney cells and A549 human lung-epithelial cells of the 9S PB1 DI virus and WT PR8 virus. (G) “DI Units” of the viruses were calculated by dividing HA units by endpoint titer. (H) Mouse bodyweight curves after infection with the indicated doses of the 9S PB1 DI virus. (I) Survival curves from H. (J) Mouse bodyweight curves after infecting mice with a sublethal dose of the 9S PB1 DI virus (500 PFU), a lethal dose of WT PR8 (20 PFU), or combination of WT PR8 and the 9S PB1 DI virus. (K) Survival curves from the infections in J. For all graphs, * represents a p-value of ≤ .05 and ** represents a p-value of ≤ .001.

The interference effect of 9S PB1 mCherry virus, while encouraging, was minimal. Since normal DI segments arise from the large-scale deletion of ORFs, we hypothesized that the protective effect of a 9S virus could potentially be enhanced by making the artificial segments smaller. We therefore designed a DI-like segment 9 based on a previously characterized PB1 DI segment reported by Saira *et al*. [20] (Fig 3A). We chose to focus this approach on the PB1-mCherry segment, as this segment showed a larger degree of protection from challenge relative to the PB2-sfGFP segment. We then rescued the 9S PB1 DI virus, now with the expectation that this virus would normally exist as a population of both 9- and 8-segmented virions (Fig 3B). Quantification of the segment packaging rates revealed that similar to the other 9S viruses, the artificial segment containing unique packaging signals (PB1 DI) is efficiently packaged while one of the segments with duplicated packaging signals (PB1 NA PS) was frequently excluded (Fig 3C). Multicycle growth curve analysis revealed that the virus grew slower relative to the parental PR8 virus by approximately the same magnitude as the other 9S viruses (Fig 3D). Analysis of titer and HA units after growth in chicken eggs, MDCK cells, and A549 cells revealed that similarly to the other 9S viruses, the 9S PB1 DI virus produced roughly 10^2^ times more non-viable progeny than WT PR8 virus (Fig 3E–3G).

As expected, the 9S PB1 DI virus was significantly attenuated *in vivo*, even slightly more so than the fluorescent 9S viruses (Fig 3H and 3I). To test the protective efficacy of the 9S DI virus, we simultaneously treated mice with 500 PFU of the DI virus together with a normally lethal dose of WT PR8. Similar to the 9S PB1 mCherry virus, the 9S PB1 DI virus was found to confer a protective effect, with weight loss occurring 24 hours later than seen in the control, and increased the rate of survival (Fig 3J and 3K).

### IAVs that require 10 genomic segments are viable and their administration can rescue infected animals from lethal viral disease

**Fig 4:**
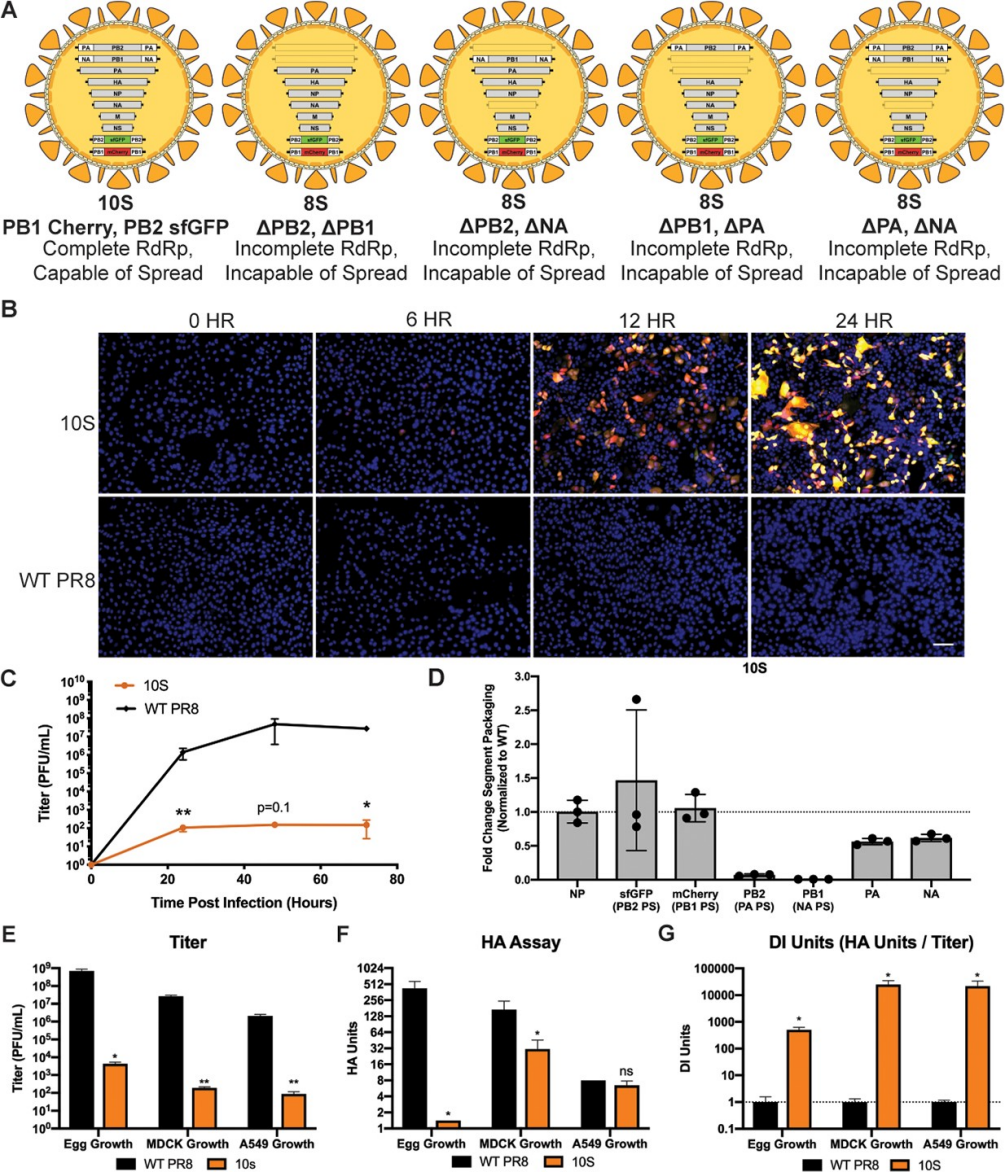
Viruses that require 10-segments for infectivity are viable and generate large amounts of DI particles. (A) Potential genomic configurations of the 10S virus assuming packaging of at least 8 genomic segments. (B) Fluorescent microscopy images of 10S or WT PR8 virus-infected MDCK cells at 6, 12, and 24 hours post-infection at an MOI of 0.1; nuclei were stained blue using DAPI staining, and the scale bar represents 100 micrometers. (C) Growth curve of the 10S and parental PR8 virus in MDCK cells infected at an MOI of 0.01. (D) qRT-PCR for the indicated genomic segments from purified 10S virions. (E) Endpoint titer 72 hours post-infection in 10-day old embryonated chicken eggs, Madin-Darby canine kidney cells (MOI = 0.01) and A549 human lung-epithelial cells (MOI = 0.01) of the 10S and WT PR8 virus. (E) HA assay of the 10S and WT PR8 virus 72 hours post-infection in 10-day old embryonated chicken eggs, Madin-Darby canine kidney cells and A549 human lung-epithelial cells. (F) The “DI Units” of the viruses were calculated by dividing HA units by endpoint titer. For all graphs, * represents a p-value of ≤ .05 and ** represents a p-value of ≤ .001.

Since the size of the DI segment did not drastically change therapeutic efficacy, we decided to increase the number of artificial segments that could potentially interfere with WT viral spread. Because our two 9S viruses were generated with non-overlapping packaging signal duplications, we tested combining these two approaches to generate a virus that required 10 genomic segments (10S) to be fully infectious. Based on our data from 9S viruses, we expected that actual 10-segmented particles would be rare and most of the particles generated would only package 8 segments (Fig 4A), however 9-segmented permutations were also a possibility (S4A Fig). Combining the two 9S strategies was successful, and we were able to rescue an IAV strain that propagated six unmodified genomic segments alongside four genetically manipulated ones. A fluorescent microscopy time-course of the 10S virus revealed that, as designed, where virus replication occurred, both of the fluorescent artificial segments were present as evidenced by the high percentage of cells expressing dual color fluorescence (Fig 4B, S4B Fig). Flow cytometry analysis of 10S infected cells also showed that the majority of cells expressing one of the fluorescent proteins were co-positive for the viral HA protein as well as the other fluorescent proteins (S4C–S4E Fig). Multicycle growth curve analysis of the 10S virus showed that it grew poorly, likely due to compounded effects of artificial segment packaging rates (Fig 4C). In order to define which segments were not efficiently packaged, we performed qRT-PCR and saw that not only were the segments with duplicated packaging signals (PB2 PA PS, and PB1 NA PS) packaged at markedly reduced rates, their WT segment counterparts of WT PA and NA were also packaged less efficiently (Fig 4D). These data suggest that 10S viruses produce a variety of defective particles with variable segment packaging. Analysis of viral titer and HA units after growth in chicken eggs, MDCK cells and A549 cells demonstrated that the 10S virus, while highly attenuated, produced a very high amount of nonviable progeny, up to 10^4^ times higher than the parental WT PR8 virus (Fig 4E–4G).

**Fig 5:**
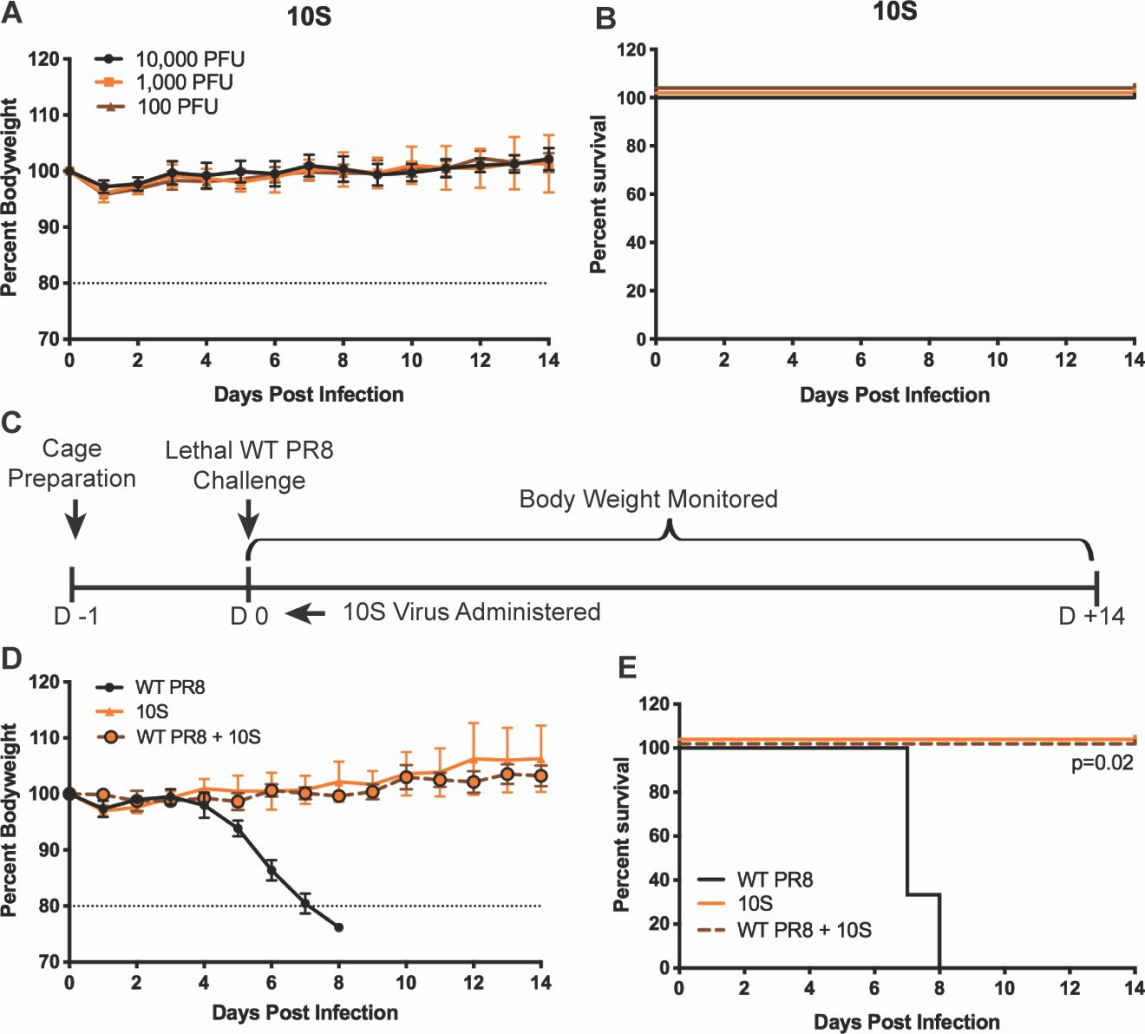
Influenza viruses that require 10 genomic segments for infectivity are highly attenuated and their administration protects mice from wild-type viral challenge. (A) Weight loss curves from infections with the indicated doses of 10S virus. (B) Survival curves from infections described in A. (C) Diagram detailing the coinfection challenge experimental design in C57BL/6J mice. (D) Bodyweight curves after infecting mice with a sublethal dose of the 10S virus (5000 PFU), a lethal dose of WT PR8 (20 PFU), or a combination of the WT PR8 and 10S viruses. (E) Survival curves from the infection groups described in D.

Next, we infected C57BL/6 mice with increasing doses of the 10S virus. As expected from the *in vitro* data, the virus was highly attenuated; animals infected with doses as high as 10^4^ PFU, which caused mortality with all of the 9S viruses, experienced no detectable body weight loss and there was no mortality (Fig 5A and 5B). While attenuation is desirable for its potential use as an antiviral, we were concerned that this limited replication *in vivo* may preclude any interfering effect against a WT IAV strain. To test the 10S interfering effect, we challenged C57BL/6 mice with 20 PFU of WT PR8 virus in combination with 5000 PFU of the 10S virus (Fig 5C). While control animals infected with only the parental PR8 lost body weight and succumbed to infection, there was no detectable morbidity or mortality when the 10S virus was co-administered (Fig 5D and 5E).

**Fig 6:**
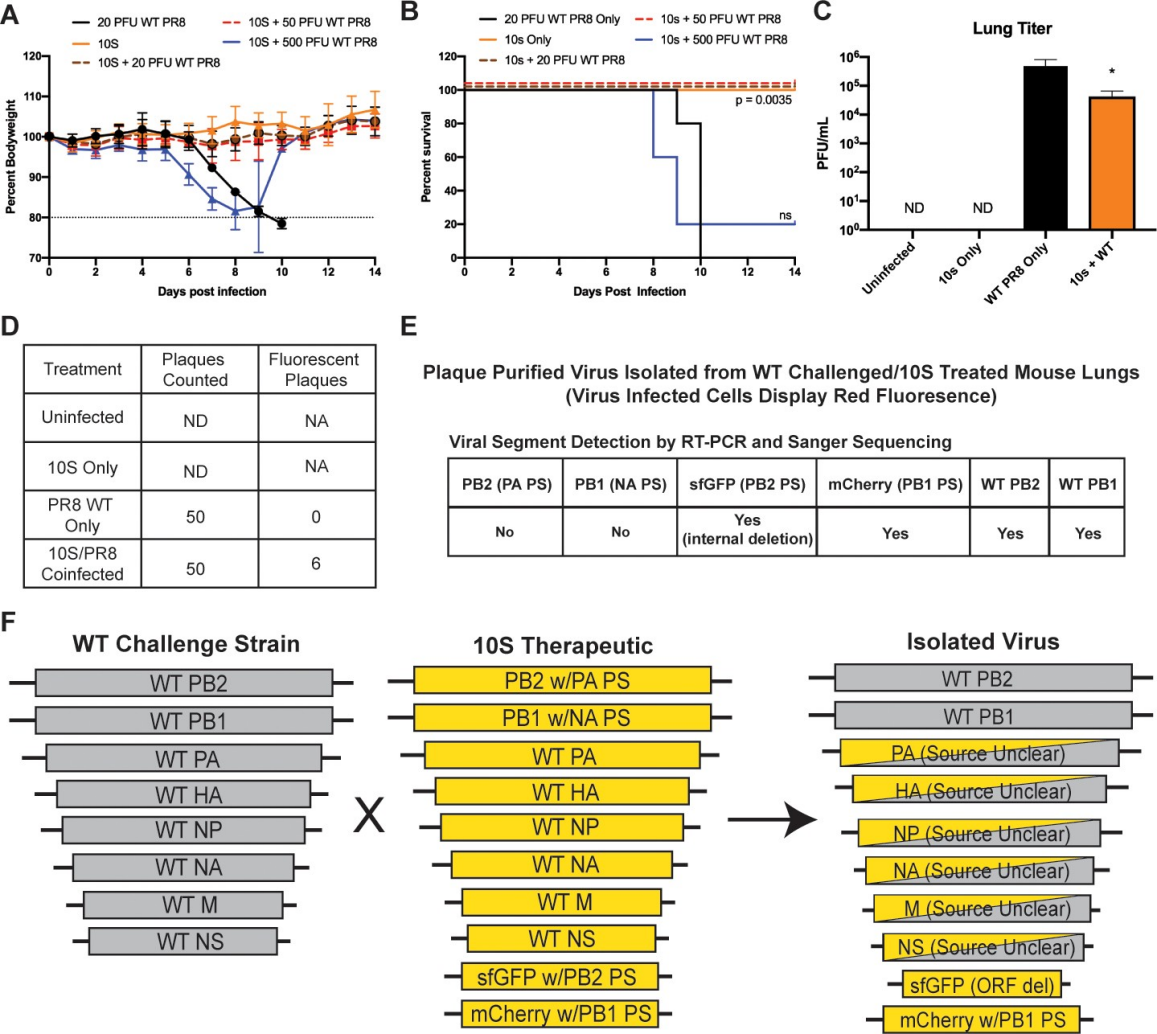
Viruses that require 10 genomic segments reassort with, and restrict the growth of, wild-type virus. (A-B) Weightloss (A) and survival (B) curves of mice treated with 5x10^3^ PFU of the 10S therapeutic virus and infected with either 20, 50, or 500 PFU of WT PR8. For panel B, the significance of the 20 and 50 PFU challenge dose was the same. (C) Viral titering of IAV from lungs of mock infected mice, mice 4 days post-infection with 5x10^3^ PFU of 10S virus alone, 20 PFU of WT PR8 alone, or coinfected with WT PR8 and the 10S virus. (D) Number of plaques assayed for fluorescence from each of the treatment group lung homogenates from C. ND-Not detected, NA-Not applicable. (E) RT-PCR and sequencing analysis of a plaque-purified virus that displayed only red fluorescence isolated from the WT PR8/10S-treated group from C. Multiple independently isolated viruses displayed the same genetic makeup. (F) Schematic of the inferred sources of the genomic segments detected in the analysis described in E. For all graphs, * represents a p-value of ≤ .05.

We next tested increasing the WT PR8 challenge dose and found that 10S co-administration still conferred complete protection at 2.5x the lethal dose, but the protective effect was effectively lost at 25x the lethal dose (Fig 6A and 6B). We hypothesized that 10S co-administration should decrease the amount of fully infectious viral particles, and analysis of viral titers from the lungs of mice challenged with the 20PFU lethal dose and treated with the 10S virus showed more than a 10-fold reduction in titer compared to infection with WT PR8 alone at 4 days post-infection (Fig 6C). Analysis of virus clones isolated from the lungs of the WT PR8/10S co-administration treatment group showed that some of the culturable viruses had co-packaged WT and 10S genomic segments; these data are consistent with reassortment being at least one of the 10S interference mechanisms (Fig 6D–6F).

**Fig 7:**
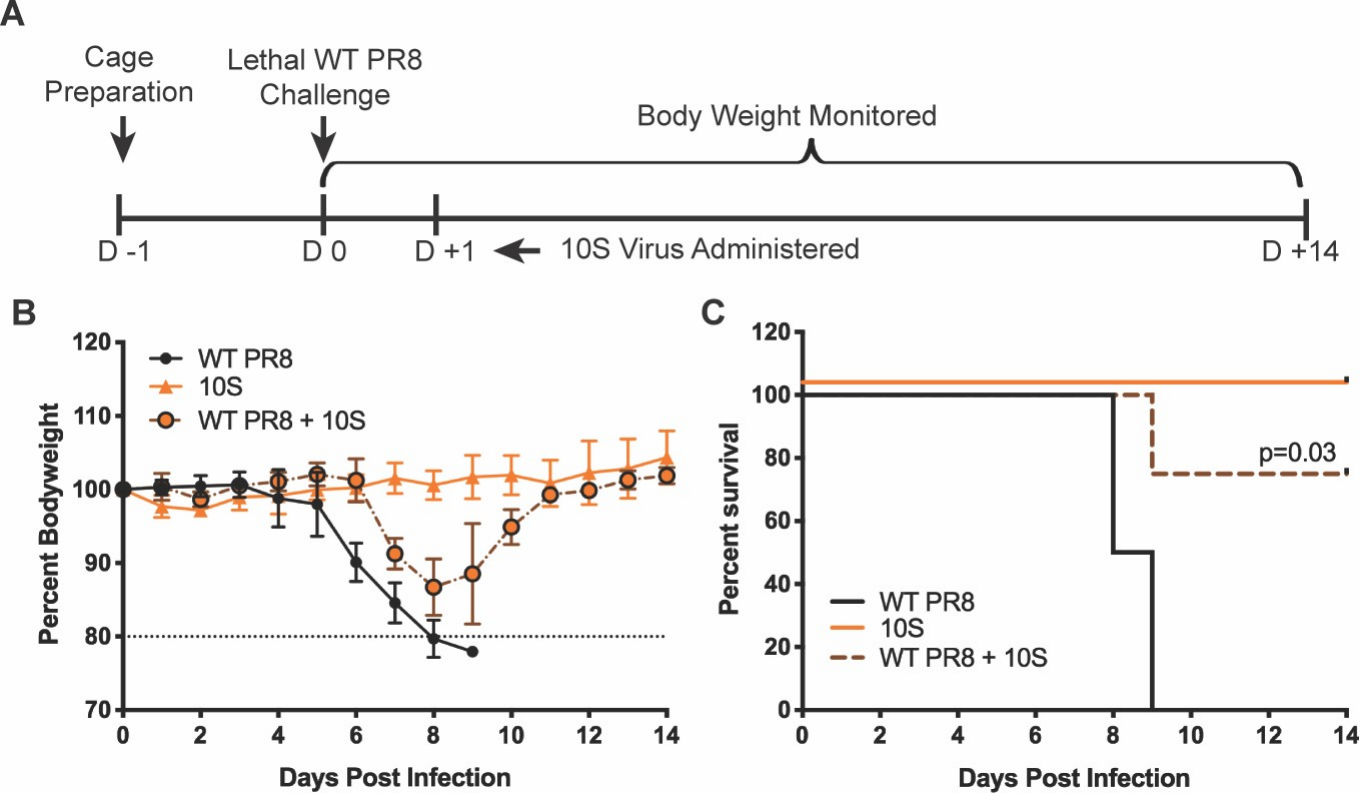
Influenza viruses that require 10 genomic segments can interfere with a lethal viral challenge when administered therapeutically. (A) Diagram of the 10S therapeutic administration experiment in C57BL/6 mice, with treatment at 24 hours post-infection with a lethal dose of WT PR8. (B) Bodyweight curves of mice treated with a dose of the 10S virus (5000 PFU), infected with a lethal dose of WT PR8 (20 PFU), or infected with WT PR8 virus followed by 10S virus administration 24 hours later. (C) Survival curves from B.

Finally, we assessed 10S virus efficacy in a therapeutic treatment application. C57BL/6 mice were infected with a lethal dose of 20 PFU of WT PR8 virus, and administered the 10S therapeutic dose 24 hours later (Fig 7A). While not completely protected from disease like with the co-administration, we observed a significant delay in the onset of disease as well as a significant increase in survival (Fig 7B and 7C). Additionally, to verify that protective effects of our PR8 based 10S virus were not restricted to PR8, we also examined 10S interference with additional strains of IAV. We observed, that in addition to interfering with replication and spread of PR8, the 10S virus could also interfere with the H3N2 X-31 virus as well as the pandemic H1N1 A/California/04/2009 viruses (S5A–S5F Fig). As expected, interference with viral spread led to a measurable decrease in cytopathic effect of the cellular monolayer (S5G Fig).

## Discussion

This research was initially started with the goal of creating a replication-competent, live-attenuated virus that would be able to propagate genomic segments capable of disrupting effective genomic packaging of a coinfecting WT virus. Our approach was mechanistically distinct from naturally occurring DI particles; the 9S and 10S platforms however, essentially mimic the concept of facilitating packaging of a defective viral segment, which then leads to the release of virions bearing incomplete viral genomes. Despite 9 and 10S viruses displaying incomplete genome packaging, delayed replication kinetics, and lower peak titers, we showed that administration of these viruses can have significant disruptive effects for the normal pathogenesis of WT IAV.

A number of new questions were raised by our development of these viruses. We tested a variety of viral genomic organizations and found that only rare combinations of viral genes and packaging signals were able to be tolerated by the virus. The rationale for why this is the case is unclear, but there are probably a number of constraints that underlie this phenomenon. First there is known to be a hierarchy of viral segment packaging [35], and thus, some segments (identified by the virus based on the packaging signals) may be less tolerant of duplication than others and lead to a disruption of the structure/assembly of the IAV genome. In line with this concept, work using seven-segmented influenza viruses has demonstrated that the requirement for different packaging signals is variable with respect to viral assembly [36]. Interestingly, this earlier work demonstrated that both the NA and PA packaging signals are not required for the packaging of the other genomic segments. Our ability to duplicate both of those packaging signals agrees with the concept that these particular packaging signals play a relatively less important role in viral assembly.

The seven-segmented virus work however, does not necessarily predict the ability of a given packaging signal to be duplicated. For example, NS packaging signals were also shown to be dispensable, yet we were unable to rescue a virus with duplicated NS packaging signals (Table 1). This discrepancy may be explained by the fact that the levels of transcription and translation of these viral segments is controlled by motifs in these specific segments [37, 38], and thus the combination of different viral ORFs and packaging signals leads to a disruption of the normal controllers of viral transcription/translation rates, negatively impacting viral fitness. This concept is somewhat supported by our data that a virus encoding the PB2 protein flanked by NP packaging signals is non-viable; NP is expressed in cells to a much higher level than PB2. When we encoded PB2 flanked by PA packaging signals however, the virus was viable, and PA and PB2 levels in the infected cell are reasonably similar [39].

Another question raised by our work is the mechanism of segment discrimination by the virus during packaging. Our qRT-PCR data on purified 9S and 10S viruses indicate that segments harboring essentially the exact same packaging signals can be discriminated from each other. In all cases, packaging of the WT segment was strongly favored (usually by more than 100x) over a modified segment which had the same packaging signal flanking a different viral ORF. It is unclear how the virus is performing this selective packaging, but we can envision a number of potential explanations. First, the packaging signals in the modified segment are not actually identical to the WT packaging signals. Because translation cannot be allowed to begin in the packaging signals (as they normally would when they overlap the ORF in a normal segment), any ATG sequences (usually two-three in a given packaging signal) are mutated to TTG. We don’t believe however, that these changes can fully explain segment discrimination, as those slightly mutated packaging signals are perfectly competent to facilitate segment packaging [40]. Other potential contributors to segment discrimination are: artificially increased UTR length, altered total segment length, or the lack (or even absence) of a required, but undefined, sequence in the ORF portion of the segment. It may be the case that 9S or 10S viruses represent an experimental system with which to answer this question and more fully define sequences that control viral genomic assembly.

Our data show that most virions package 8 segments even when segments with duplicated packaging signals are required for complete infectivity. It is important to note that our data do not allow us to define how often a fully infectious event is derived from true packaging of a 9^th^ or 10^th^ genomic segment in a viral particle or from co-infection of particles with incomplete genomes. We believe that both of these possibilities likely occur, with virions harboring extra segments potentially playing a more important role early in infection when the number of viral particles per cell is low. If true that some viral particles can functionally package extra genomic segments, it is unclear how they would be organized within the capsid. It is well accepted that IAVs package their segments in a “pinwheel” or 7+1 conformation, wherein a single segment, most likely one of the polymerase segments based on its size, is packaged in the center with the remaining seven segments arranged around it in a circular shape [41]. Understanding how the addition of one or two segments could alter this structure could lead to a better understanding of IAV packaging in general. Along these same lines, it is currently unclear how much nucleic acid an influenza virus particle can hold. Other published IAV engineering strategies have significantly increased the length of genomic segments [42–45], and potentially increasing the number of genomic segments raises important questions about not only the maximum amount of genetic material tolerated, but also what defines that limit. Answering these questions becomes especially relevant when considering the potential for utilizing influenza viruses as viral vectors [46].

Finally, the fact that 9S and 10S viruses can interfere with WT viral propagation strongly supports the notion that cellular coinfection is a common occurrence *in vivo*. Despite the fact that IAV particles package all eight genomic segments the majority of the time [47, 48], a relatively small percentage of viral particles are fully infectious [49]. Recent work has suggested that coinfection between incompletely infectious particles may be a frequent occurrence that not only allows viral reassortment [50], but also plays a critical role in normal viral spread across infected tissues [51]. Since 9S and 10S interference with WT viral packaging are dependent on coinfection with WT viruses, we not only favor this model, but propose that even distinct viral infections that begin at different times are also subject to this coinfection phenomenon. It is worth noting however, that the protective effects of 9S and 10S viruses are not necessarily mediated exclusively by interfering with genomic segment packaging, as indirect effects of defective particles such as innate immune activation could also contribute to protection from IAV challenge.

In summary, we have successfully defined genomic architectures that allow influenza viruses to propagate up to two additional, artificial segments. Our work suggests that not all IAV packaging signals are amenable to manipulations such as duplication, and that the particular characteristics of an artificial viral segment are not as important to its interfering effect as the absolute number of segments that can disrupt productive genomic packaging. Continued development of the 10-segmented replication-competent IAV platform may lead to a novel class of therapeutics that can be easily manufactured, safely administered, and display protective efficacy against viruses that have evolved resistance to other antiviral therapies.

## Materials and methods

### Ethics statement

All animal procedures were carried out in compliance with the Duke University IACUC approved protocol number A189-18-08. Duke University maintains an animal program that is registered with the United Stated Department of Agriculture Animal Welfare Act (#863), assured through the National Institutes of Health Policy on Humane Care and Use of Laboratory Animals (#D16-00123, A3195-01), and accredited by AAALAC International (#363). Animals were monitored daily for the following: respiratory rate, ambulating difficulty, ruffled fur, lack of grooming, restlessness, reluctance to move, and bodyweight loss. Humane endpoints were primarily based on bodyweight loss, and defined as ≥ 20% of the starting bodyweight. The primary euthanasia method used was CO_2_ asphyxiation, followed by bilateral thoracotomy as a secondary method. SPF embryonated chicken eggs were purchased from Charles River Labs and incubated in the laboratory for viral stock amplification. Eggs were injected with virus 8–10 days post-fertilization and incubated until a maxim age 13 days post-fertilization.

### Animal infections

Eight to 10-week-old C57BL/6 mice were purchased from Jackson Laboratories and maintained at Duke University animal facilities. For all experiments, a sample size of at least 3 mice per group were used. Prior to infection, mice were anesthetized with a 100-microliter injection of a ketamine-xylazine mixture. Mice were weighed and tail-marked, and 40 microliters of virus diluted in pharmaceutical-grade PBS was administered intranasally. Mice were subsequently weighed daily, and euthanized once the predetermined humane endpoint was reached. Therapeutic doses of 9S and 10S viruses were ½ of the highest dose that caused only mild disease in mice when administered alone.

### Cell culture

Madin-Darby canine kidney (MDCK) cells, from the American Type Culture collection (ATCC), were grown in minimal essential medium (MEM) supplemented with 10% fetal bovine serum, HEPES, NaHCO3, GlutaMAX, and penicillin-streptomycin. Human embryonic kidney 293T cells and human alveolar basal adenocarcinoma epithelial (A549) cells (from the ATCC) were grown in Dulbecco’s modified Eagle’s medium (DMEM) supplemented with 10% fetal bovine serum, GlutaMAX, and penicillin-streptomycin. All cells were cultivated at 37°C, at a 5% CO_2_ content, in the humidity controlled Heracell VIOS 160i Thermo Scientific incubators.

### Cloning and rescue of 9S and 10S viruses

Recombinant influenza viruses were generated as previously described by use of the ambisense pDZ rescue plasmid system [43]. The 9S PB1 mCherry segment was generated similarly to the construct described in [34], replacing GFP with mCherry. Briefly, the mCherry fluorescent protein coding sequence, preceded by a Kozak sequence (gccacc), was cloned into a PR8 PB1 packaging vector using PCR and subsequent NEBuilder HiFi DNA Assembly reaction. The PB1 packaging vector consisted of nucleotides 1–146 of the 5’-most PB1 sequence (with all ATG start sites mutated) followed by an EcoRV site. A PmeI restriction site separated the 3’-most PR8 PB1 sequence of nucleotides 2189–2341. The PB1 coding sequence (nucleotides 25–2298), with a 5’ Kozak sequence and was cloned into a PR8 NA packaging vector. The PB1 coding sequence 5’-most 75 nt and 3’-most 81 nt were silently mutated to remove packaging signal activity. The NA packaging vector consisted of nucleotides 1–173 of the 5’-most PR8 NA sequence (with all ATG start sites mutated) followed by an EcoRV site. A PmeI sequence separated the subsequent 3’-most NA sequence of nucleotides 1205–1413. Packaging vectors and primers were synthesized as ordered through Integrated DNA Technologies, Inc.

The 9S PB2 sfGFP segment was generated as follows: The sfGFP fluorescent protein coding sequence, preceded by a 5’ Kozak sequence, was cloned into a PR8 PB2 or Wyo/03 PB2 packaging vector. The PB2 packaging vector for both PR8 and Wyo/03 consisted of nucleotides 1–152 of the 5’-most PB2 sequence (with all ATG start sites mutated to TTG) followed by a NheI site. An XhoI sequence separated the 3’-most PB2 sequence of nucleotides 2178–2341. The PB2 coding sequence (nucleotides 28–2307) with a 5’ Kozak sequence was cloned into a PR8 PA or Wyo03 PA packaging vector. The PB2 coding sequence 5’-most 30 nt and 3’-most 85 nt for PR8, and the 5’-most 60 nt and 3’ most 51 nt for Wyo03 were silently mutated to remove packaging signal activity. The PA packaging vector for both PR8 and Wyo/03 consisted of nucleotides 1–129 of the 5’-most PA sequence (with all ATG start sites mutated to TTG) followed by an EcoRV site. A PmeI sequence separated the subsequent 3’-most PA sequence of nucleotides 2050–2233.

The 9S PB1 DI segment is based upon characterization of INS002, as described in [20]. DNA was synthesized via Integrated DNA Technologies, Inc. in which the aforementioned PB1 packaging vector contained PR8 PB1 nucleotides 146–190 followed by nucleotides 2094–2188. In the 5’-most region of this construct, all ATG start codons were mutated to prevent undesired translation initiation.

9S and 10S viruses were generated as previously described [34, 43]. Briefly, the specific combination of artificial and wildtype plasmid segments were transfected into low-passage 293T cells using the Mirus Trans-IT LT1 reagent. Rescue supernatant was collected after 48 hours of incubation and injected into 10-day-old embryonated chicken eggs purchased from Charles River Laboratories, Inc. Eggs were allowed to incubate for 72 hours prior to collection of allantoic fluid and subsequent plaque-purification of successfully rescued virus. Stocks of concentrated 10S virions were prepared using a 30% sucrose cushion for 1 h at 25,700 rpm on the Sorvall TH-641 swinging bucket rotor.

### Viral titering

Allantoic fluid from chicken eggs or supernatant from MDCK and A549 cells was collected following infection, and viral titer was determined via standard plaque assay procedures on MDCK cells [42]. Briefly, cells were incubated with virus dilutions before removing the virus and applying the agar overlay. Cells were then incubated at 37°C for 48–72 hours before being fixed in 4% paraformaldehyde (PFA) in phosphate-buffered saline (PBS) for at least 4 h. Plaques were stained with polyclonal mouse serum from PR8-infected mice and an anti-mouse IgG horseradish peroxidase (HRP)-conjugated sheep antibody (GE Healthcare) was used as a secondary antibody. Assays were developed with True Blue reagent and individual plaques were counted (only wells with greater than 3 plaques were used for the calculation of endpoint titer).

### Viral growth curves

For each growth curve, MDCK cells, A549 cells or 10-day old embryonated chicken eggs were infected with the indicated virus. Supernatant or eggs were collected at 24, 48, and 72 hours post-infection. Aliquots of allantoic fluid or cell supernatant were immediately frozen at -80°C to be thawed for use in HA assays and standard plaque assay procedures. All experiments were conducted in biological triplicate.

### Hemagglutination (HA) assay

Hemagglutination (HA) assays were performed by diluting virus-containing allantoic fluid or cell supernatant in cold PBS. 50 microliters of chicken blood diluted 1:80 in cold PBS was mixed with each sample and incubated at 4°C overnight prior to scoring. Turkey blood was used at a dilution of 1:40 for performing HA assays on all samples containing the A/California/04/09 virus.

### DI unit calculation

Defective Interfering or “DI” Units were calculated by normalizing a virus’s HA score and endpoint titer to that of WT PR8. These normalized values were then averaged, and the HA score was divided by its normalized, averaged endpoint titer.

### Microscopy time course

MDCKs were infected for 1 hour at an MOI of 0.1 with either the 9S PB1 mCherry, 9S PB2 sfGFP, 10S, or WT PR8 virus diluted in PBS/BSA. Following the incubation period, the infection medium was removed and cells were placed in complete medium supplemented with 1:1000 diluted TPCK trypsin. At the indicated time after infection, MDCK cell medium was removed and replaced with 1 ml of warm PBS. Cells were incubated with Hoechst stain (1 microliter/ml of PBS) to allow for the staining of nuclei, and imaging was performed on the Zoe fluorescent cell imager (Bio-Rad) using the same gain, exposure and zoom settings for all images taken. Images were then processed with ImageJ (NIH).

### Flow cytometry

MDCK cells were given a single-cycle infection (no TPCK trypsin was added) with either WT PR8 virus, the 8S NS1-ZsGreen control virus (constructed as described in reference [52] replacing GFP with ZsGreen), the 9S PB2 sfGFP virus, the 9S PB1 mCherry virus, or the 10s virus at an MOI of 0.1. 24 hours post-infection, cells were collected and fixed with 1% PFA for 15 minutes prior to being spun down and resuspended in fresh PBS with 1% Bovine serum albumin (BSA). Cells were then incubated for 1 hour with 1:500 dilution of the monoclonal antibody PY102 (an antibody specific for the HA from the A/Puerto Rico/08/1934 strain) [42]. After this incubation, cells were washed and then stained for 2 hours with 2 ug/mL of anti-mouse APC conjugated secondary antibody (Life technologies cat A865). All cells were run on a Fortessa X-20 (BD) machine with standard laser and filter combinations and data was visualized and processed with the Flowjo software.

### Harvesting and virus titer from mouse lungs

Eight to 10-week old C57BL/6 mice were infected in the same manner as described above. Four days post-infection, mice were collected and given a lethal dose of ketamine-xylazine (400 uL). Once sufficiently anesthetized, a cervical dislocation was performed before removing the whole lung and trachea from the mice. Lung samples were then homogenized in 1 mL of PBS using the Benchmark BeadBlaster 24 Microtube Homogenizer. Homogenized samples were then spun to pellet the insoluble pieces of lung and the supernatant was collected, aliquoted and frozen at -80C. Lung supernatants were then thawed and titered using the plaque assay protocol listed above.

### Viral isolation and taqman assays

All viruses were first amplified in 10-day old embryonated chicken eggs for 72 hours. Allantoic fluid was then collected and filtered through a 0.45 um filter to remove any contaminating infected chicken cells. Viral RNA was then extracted from the filtered allantoic fluid using standard Trizol extraction procedures [42]. Reactions were prepared using the EXPRESS One-Step Superscript qRT-PCR kit (ThermoFisher Scientific (cat 11781200), and performed on a StepOnePlus Real-Time PCR System with the listed probes in S1 Table. Relative abundance was calculated using CT values for each sample.

### *In vitro* coinfection experiments

MDCK cells were either singly infected with WT PR8 (.01 MOI) only, the H3N2 X-31 reassortant (.01 MOI) only, the pandemic H1N1 A/California/4/2009 (.01 MOI) only, or the 10S virus (.01 MOI) only; or coinfected with Cal09 (.01 MOI) and PR8 (.075 MOI), X-31 (.01 MOI) and PR8 (.075 MOI), Cal09 (.01 MOI) and the 10S virus (.075 MOI), X-31 (.01 MOI) and the 10S virus (.075 MOI), or PR8 (.01 MOI) and the 10S virus (.075 MOI) for 1 hour. Following the incubation period, infection medium was aspirated and replaced with complete medium supplemented with 1:1000 TPCK trypsin. At 24, and 48 hours post-infection, 100 uL of supernatant was collected and immediately used to perform an HA assay. At 72 hours the remaining supernatant was collected and used to perform both HA and plaque assays, while cells were immediately washed 1x with PBS and then incubated in 1 mL of fresh PBS containing 1:10,000 Hoechst dye and imaged on the Zoe fluorescent cell imager (Bio-Rad). All images were processed with ImageJ (NIH).

### Statistical analyses

Comparisons between treatment groups were performed using Prism 8 software (Graphpad). For the animal survival experiments, a Mantel-Cox test was performed to assess the effects of the 9S and 10S treatment, and comparisons were made to the lethal control challenge with WT PR8. All other comparisons in the study were performed using a two-tailed, unpaired, students t-test. In instances when multiple comparisons were being performed (i.e. timepoints over the course of a viral growth curve), a Holm-Sidak correction was applied. For all graphs unless the p-value is specifically indicated, * represents a p-value of ≤ .05 and ** represents a p-value of ≤ .001, and “ns” indicates the groups were not significantly different from each other.

## Supporting information

S1 Table The sequences of the primers and probes used for viral segment qRT-PCR assays. (DOCX)

S1 Fig Diagrams of the artificial viral segments tested in this study. (A) Design of PB1 ORF flanked by NA packaging signals. (B) Design of mCherry ORF flanked by PB1 packaging signals. (C) Design of PB2 ORF flanked by NP packaging signals. (D) Design of sfGFP ORF flanked by PB2 packaging signals. (E) Design of the HA ORF flanked by NS packaging signals. (F) Design of sfGFP ORF flanked by HA packaging signals. (G) Design of PB2 ORF flanked by PA packaging signals. (H) Design of M1 ORF flanked by HA packaging signals. (I) Design of the zsGreen (splice site) M2 ORF flanked by M packaging signals. (J) Design of the NS1 ORF flanked by NA packaging signals. (K) Design of the mCherry (splice site) NEP ORF flanked by NS packaging signals. For all diagrams, the indicated regions define the number of nucleotides. Dark grey regions represent silently mutagenized regions of the viral ORF.
(TIF)

S2 Fig Flow cytometry analysis of fluorescence and viral protein co-positivity. (A) The percentage of HA (APC) positive cells detected by flow cytometry 24 hours after a single-cycle infection that are also FITC positive after infection with the NS1-ZsGreen 8S control virus. (B) The percentage of FITC positive cells that are also HA (APC) positive 24 hours after a single-cycle infection with the NS1-ZsGreen 8S control virus. The 8S reporter virus was generated essentially as described by Perez *et al*. [52] replacing GFP with ZsGreen. (C) The percentage of HA (APC) positive cells detected by flow cytometry 24 hours after a single-cycle infection that are also FITC positive after infection with the 9S sfGFP PB2 virus. (D) The percentage of FITC positive cells that are also HA (APC) positive 24 hours after a single-cycle infection with the 9S sfGFP PB2 virus. (E) The percentage of HA (APC) positive cells detected by flow cytometry 24 hours after a single-cycle infection that are also PE Texas Red positive after infection with the 9S mCherry PB1 virus. (F) The percentage of PE Texas Red positive cells detected by flow cytometry 24 hours after a single-cycle infection that are also HA (APC) positive after infection with the 9S mCherry PB1 virus. For A&B, C&D, and E&F, the same flow cytometry sample was gated using reciprocal gating strategies to show marker co-positivity was independent of which gate was applied first. The bar graphs represent three independent samples derived from the gating strategy to their immediate left. All infections were done at an MOI of 0.1 without TPCK trypsin.
(TIF)

S3 Fig 9-segmented viruses can be generated in an H3N2 A/Wyoming/03/2003 background. (A) Schematic of the segments used to generate the 9S PB2 sfGFP H3N2 virus in the A/Wyoming/03/2003 background. (B) Images of infected cells 48 hours post-infection with mock, WT Wyo/03 and the Wyo/03 9S PB2 sfGFP virus. (C-E) Analysis of viral titer, hemagglutination units, and the calculated relative DI units of the 9S Wyo/03 virus. (F) qRT-PCR analysis of viral genomic segment packaging from purified Wyo/03 9S viral particles.
(TIF)

S4 Fig 10S virus genomic configurations and quantification of fluorescence and viral protein co-positivity. (A) Schematic of the possible 9-segmented viruses missing one of the segments possessing duplicated packaging signals. (B) Microscopy images displaying the blue, green, and red channels as well as a composite image of MDCK cells 24 hours post-infection with the 10S virus. (C) Flow cytometry plots displaying the APC positive, APC + FITC double positive, and APC + FITC + PE-Texas Red triple positive cells alongside a graph quantifying the percentage of triple positive cells after 10S infection. (D) Flow cytometry plots displaying the FITC positive, FITC + PE-Texas Red double positive, and FITC + PE-Texas Red + APC triple positive cells alongside a graph quantifying the percentage of triple positive cells after 10S infection. (E) Flow cytometry plots displaying the PE-Texas Red positive, PE Texas Red + APC double positive, and PE Texas Red + APC + FITC triple positive cells alongside a graph quantifying the percentage of triple positive cells after 10S infection. For C, D, and E, the same flow cytometry sample was gated using the indicated gating strategies to show marker co-positivity was independent of which gate was applied first. The bar graphs represent three independent samples derived from the gating strategy to their immediate left. All infections for the flow-cytometry experiments were performed at an MOI of 0.1 without TPCK trypsin.
(TIF)

S5 Fig 10-segmented viruses are capable of interfering with multiple strains of IAV *in vitro*. (A) HA units at 24, 48, and 72 hours post-infection of MDCK cells with either WT PR8 or coinfected with WT PR8 and the 10S virus. (B) HA units at 24, 48, and 72 hours post-infection of MDCK cells with either the X-31 reassortant or coinfected with X-31/WT PR8 or X-31/10S virus. (C) HA units at 24, 48, and 72 hours post-infection of MDCK cells with Cal09 or coinfected with Cal09/WT PR8 or Cal09/10S virus. (D) Endpoint titer 72 hours post-infection of MDCK cells with either WT PR8 or coinfected with WT PR8 and the 10S virus. (E) Endpoint titer 72 hours post-infection of MDCK cells with either the X-31 reassortant or coinfected with X-31/WT PR8 or X-31/10S virus. (F) Endpoint titer 72 hours post-infection of MDCK cells with Cal09 or coinfected with Cal09/WT PR8 or Cal09/10S virus. (G) Microscopy images of the cell monolayer before and after infection with either WT PR8, the H3N2 X-31 reassortant, and the pandemic H1N1 A/California/04/2009 virus as compared to after coinfection with either the 10S virus or WT PR8. For all graphs, error bars indicate the SEM, * represents a p-value of ≤ 0.05, and “ns” indicates that there was no significant difference.
(TIF)
