# Supplementary material for: Influenza viruses that require 10 genomic segments as antiviral therapeutics
Source: PLoS Pathog. 2019 Nov 15;15(11):e1008098. doi: 10.1371/journal.ppat.1008098 (PMC6881065; doi:10.1371/journal.ppat.1008098)
Supplement: S1 Table — (DOCX) [file ppat.1008098.s001.docx]

**S1 Table.** The sequences of the primers and probes used for viral segment qRT-PCR assays.

| Target  ORF | Primer 1 | Primer 2 | Probe |
| --- | --- | --- | --- |
| PR8 HA | CCATCTATCATTCCAGTCCATCC | ACACTCCGTCCATTCAATCC | CCCCTTCAATAAAACCGGCAATGGC |
| PR8 NP | GAGTGACATCAAAATCATGGCG | CGTTCTCCATCAGTCTCCATC | AGGCACCAAACGGTCTTACGAACA |
| PR8 PB1 | TGTTGCCAATTTCAGCATTGG | AACTCCAATACTCATGTCCGC | TCGTTGATCCCAGACACCCCAAAA |
| PR8 PB2 | CAGCAAAATGGGTGTAGATGAG | GTTTCTCTGTTCCCTGTGTTTC | ACATTTCCTCGTTGGTCCCGGATTC |
| PR8 PA | CACATTTTCTCGTTCACTGGG | CTGACGAAAGGAATCCCAGAG | AGTCTGGTTTGGATCCTAGCCCTGC |
| PR8 NA | GTTCCTGTTACCCTGATACCG | TCACCGAAAACCCCACTG | TGATCGAAGACACCCATGGCCG |
| sfGFP | ATGTCGAGGATGGAAGTGTTC | AAGACGGATTGGGTTGATTAGG | CCAATGGGTGTGTTCTGCTGATAATGGT |
| mCherry | TTACAAAGTCAAGCTCCGAGG | TCGCCTTTAAGAGCACCATC | TCCCAAGTGATGGACCAGTCAATGC |
| WYO HA | CTCAGTGTGATGGCTTCCAA | GTGACCTAAGGGAGGCATAATC | AACGCAGCAAAGCCTACAGCAAC |
| WYO NP | GTGAGCAACTGATCCTCTCAATA | TGGTGGATCAAGTGAGAGAAAG | TCGGAACCCAGGAAATGCTGAGAT |
| WYO PB2 | CTTGTTCTTCAGTCGGGTTCT | CAGCAGATCCACTAGCATCTTTA | CGGGACAAGGATGGTGGACATTCT |
| WYO PA | GCATTGAGCAGGGCAGTATTA | TGCAAGCATGAGGAGGAATTAT | TTCACAGCAGAGGTGTCCCATTGT |
