# Supplementary figures and images for: Influenza viruses that require 10 genomic segments as antiviral therapeutics
Source: PLoS Pathog. 2019 Nov 15;15(11):e1008098. doi: 10.1371/journal.ppat.1008098 (PMC6881065; doi:10.1371/journal.ppat.1008098)

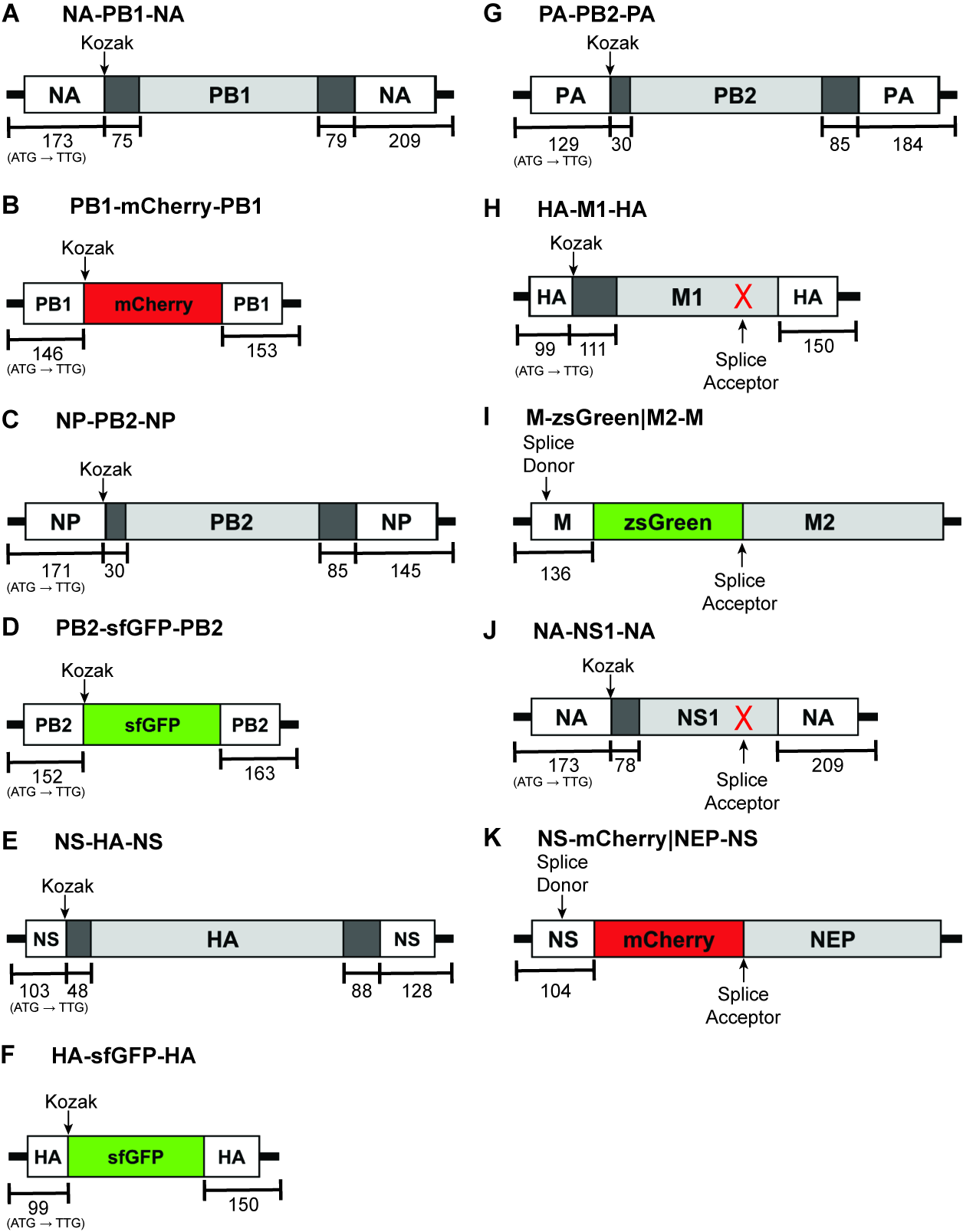

Supplement: S1 Fig — (A) Design of PB1 ORF flanked by NA packaging signals. (B) Design of mCherry ORF flanked by PB1 packaging signals. (C) Design of PB2 ORF flanked by NP packaging signals. (D) Design of sfGFP ORF flanked by PB2 packaging signals. (E) Design of the HA ORF flanked by NS packaging signals. (F) Design of sfGFP ORF flanked by HA packaging signals. (G) Design of PB2 ORF flanked by PA packaging signals. (H) Design of M1 ORF flanked by HA packaging signals. (I) Design of the zsGreen (splice site) M2 ORF flanked by M packaging signals. (J) Design of the NS1 ORF flanked by NA packaging signals. (K) Design of the mCherry (splice site) NEP ORF flanked by NS packaging signals. For all diagrams, the indicated regions define the number of nucleotides. Dark grey regions represent silently mutagenized regions of the viral ORF. (TIF) [file ppat.1008098.s002.tif]

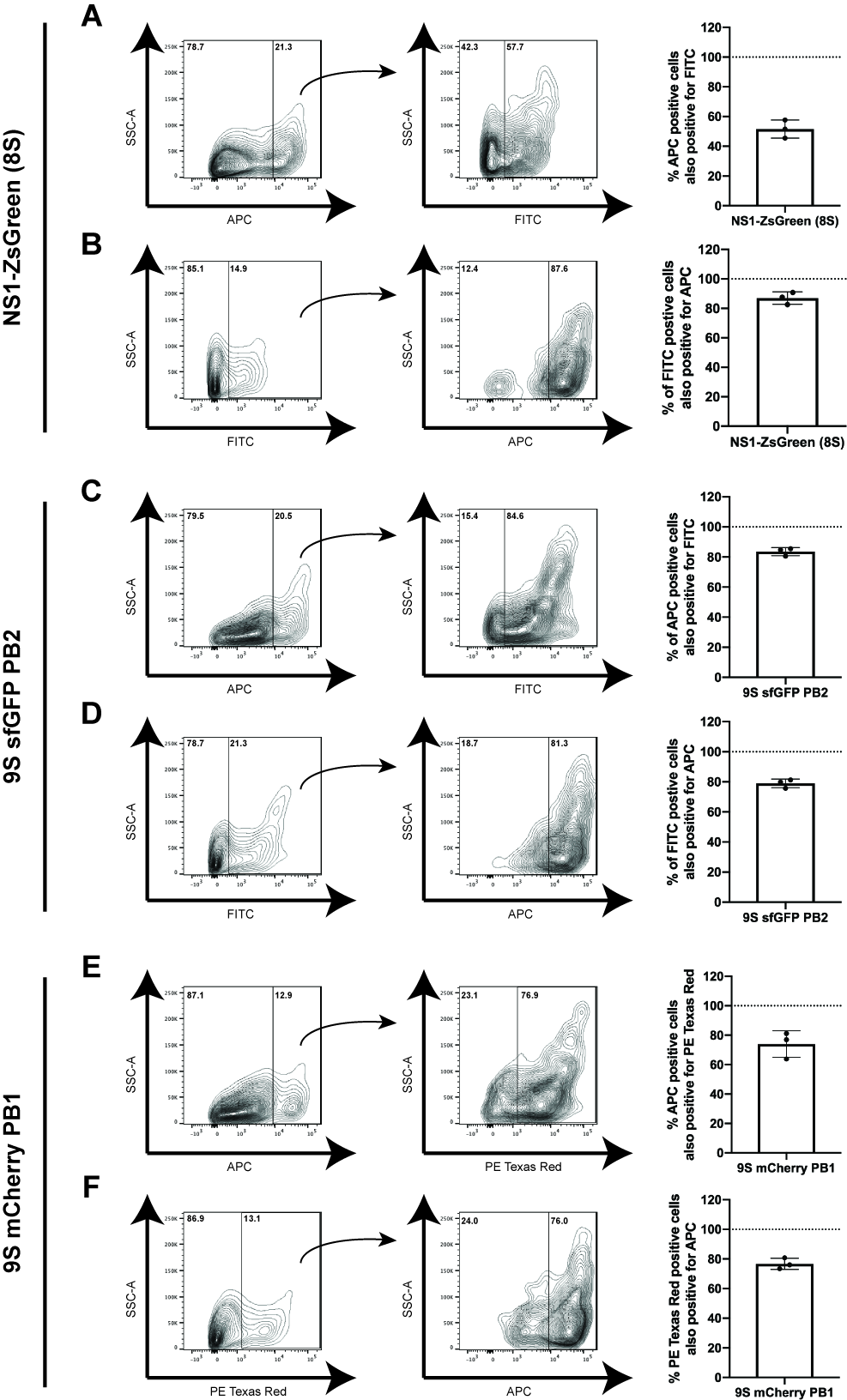

Supplement: S2 Fig — (A) The percentage of HA (APC) positive cells detected by flow cytometry 24 hours after a single-cycle infection that are also FITC positive after infection with the NS1-ZsGreen 8S control virus. (B) The percentage of FITC positive cells that are also HA (APC) positive 24 hours after a single-cycle infection with the NS1-ZsGreen 8S control virus. The 8S reporter virus was generated essentially as described by Perez et al. [52] replacing GFP with ZsGreen. (C) The percentage of HA (APC) positive cells detected by flow cytometry 24 hours after a single-cycle infection that are also FITC positive after infection with the 9S sfGFP PB2 virus. (D) The percentage of FITC positive cells that are also HA (APC) positive 24 hours after a single-cycle infection with the 9S sfGFP PB2 virus. (E) The percentage of HA (APC) positive cells detected by flow cytometry 24 hours after a single-cycle infection that are also PE Texas Red positive after infection with the 9S mCherry PB1 virus. (F) The percentage of PE Texas Red positive cells detected by flow cytometry 24 hours after a single-cycle infection that are also HA (APC) positive after infection with the 9S mCherry PB1 virus. For A&B, C&D, and E&F, the same flow cytometry sample was gated using reciprocal gating strategies to show marker co-positivity was independent of which gate was applied first. The bar graphs represent three independent samples derived from the gating strategy to their immediate left. All infections were done at an MOI of 0.1 without TPCK trypsin. (TIF) [file ppat.1008098.s003.tif]

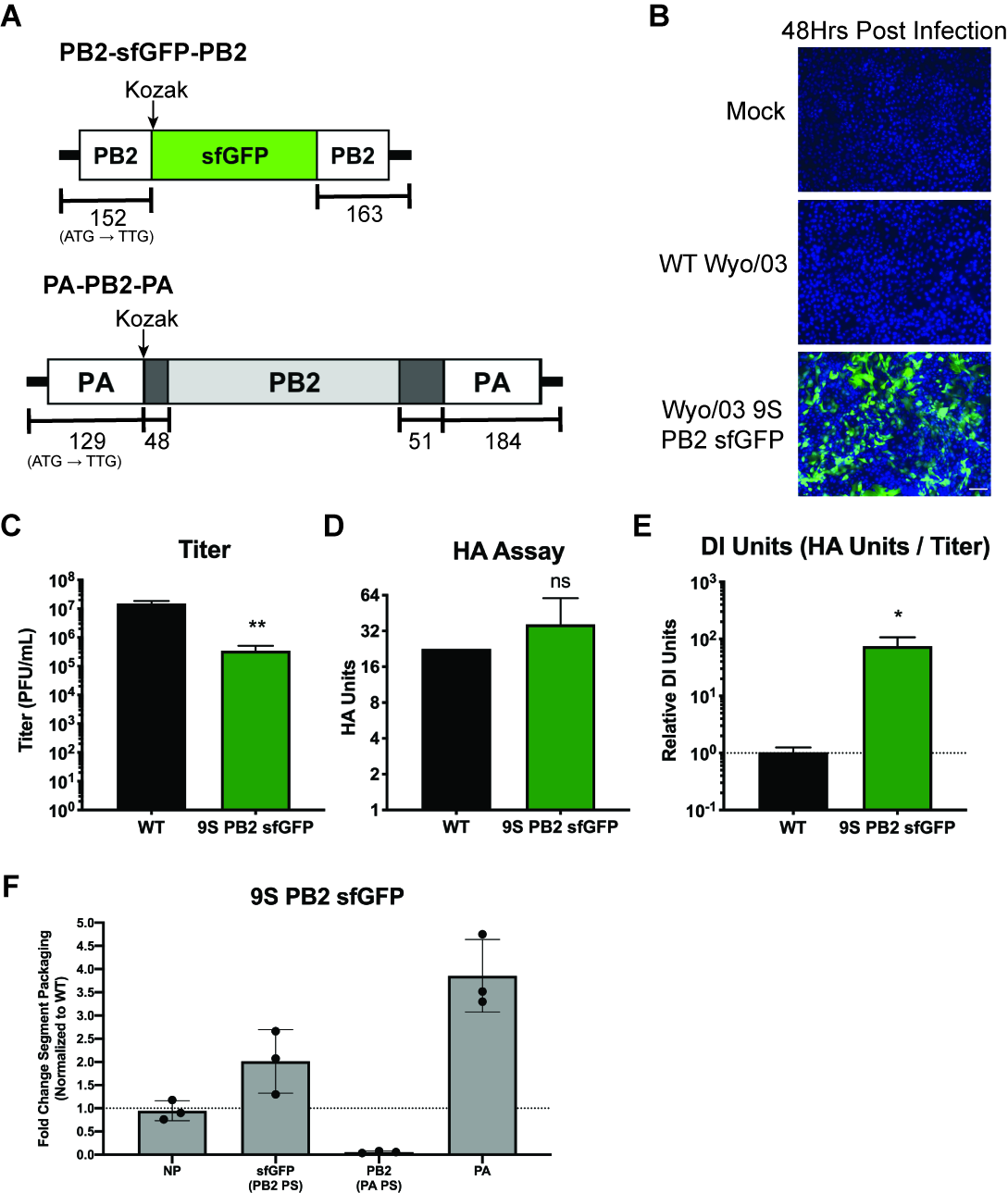

Supplement: S3 Fig — (A) Schematic of the segments used to generate the 9S PB2 sfGFP H3N2 virus in the A/Wyoming/03/2003 background. (B) Images of infected cells 48 hours post-infection with mock, WT Wyo/03 and the Wyo/03 9S PB2 sfGFP virus. (C-E) Analysis of viral titer, hemagglutination units, and the calculated relative DI units of the 9S Wyo/03 virus. (F) qRT-PCR analysis of viral genomic segment packaging from purified Wyo/03 9S viral particles. (TIF) [file ppat.1008098.s004.tif]

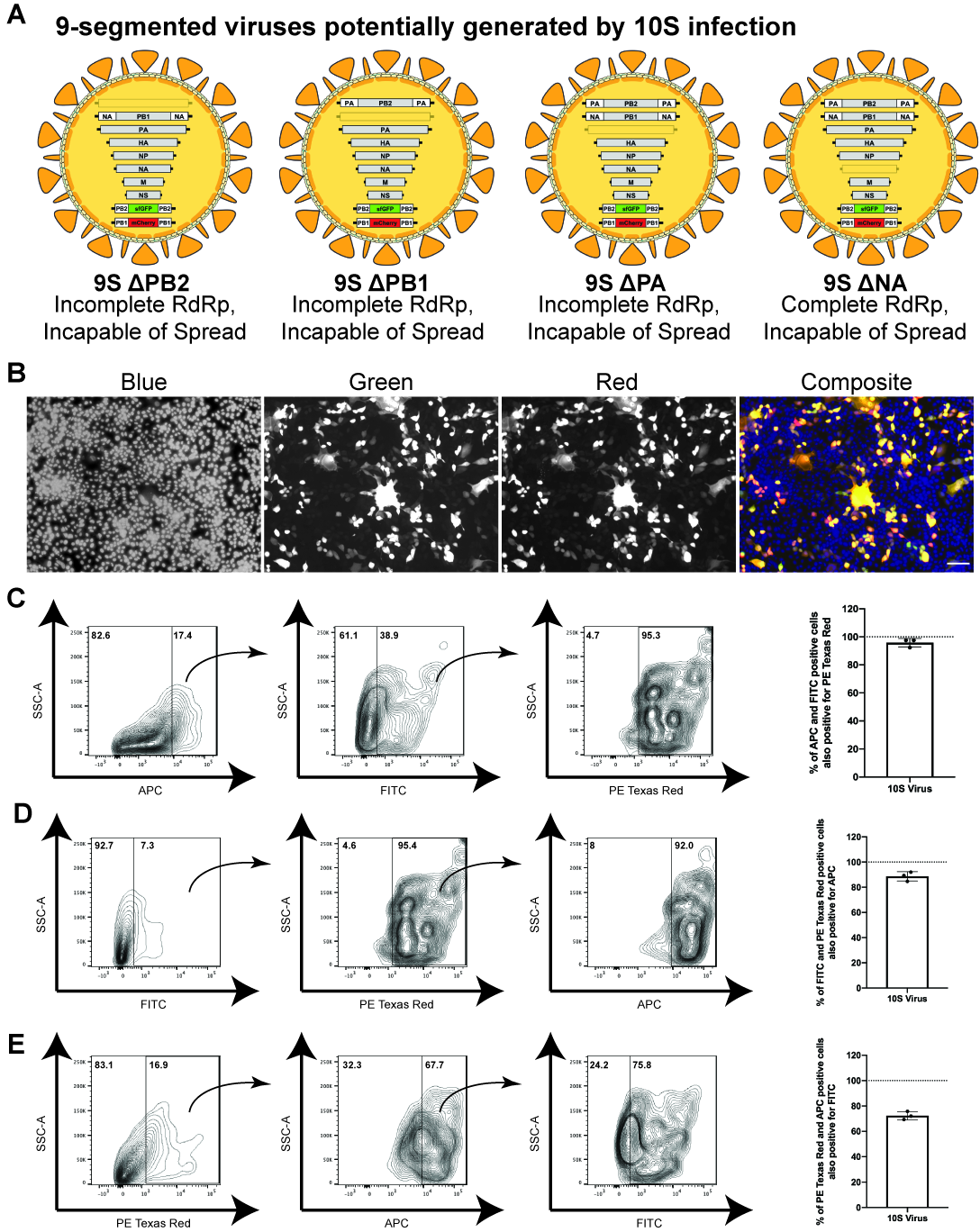

Supplement: S4 Fig — (A) Schematic of the possible 9-segmented viruses missing one of the segments possessing duplicated packaging signals. (B) Microscopy images displaying the blue, green, and red channels as well as a composite image of MDCK cells 24 hours post-infection with the 10S virus. (C) Flow cytometry plots displaying the APC positive, APC + FITC double positive, and APC + FITC + PE-Texas Red triple positive cells alongside a graph quantifying the percentage of triple positive cells after 10S infection. (D) Flow cytometry plots displaying the FITC positive, FITC + PE-Texas Red double positive, and FITC + PE-Texas Red + APC triple positive cells alongside a graph quantifying the percentage of triple positive cells after 10S infection. (E) Flow cytometry plots displaying the PE-Texas Red positive, PE Texas Red + APC double positive, and PE Texas Red + APC + FITC triple positive cells alongside a graph quantifying the percentage of triple positive cells after 10S infection. For C, D, and E, the same flow cytometry sample was gated using the indicated gating strategies to show marker co-positivity was independent of which gate was applied first. The bar graphs represent three independent samples derived from the gating strategy to their immediate left. All infections for the flow-cytometry experiments were performed at an MOI of 0.1 without TPCK trypsin. (TIF) [file ppat.1008098.s005.tif]

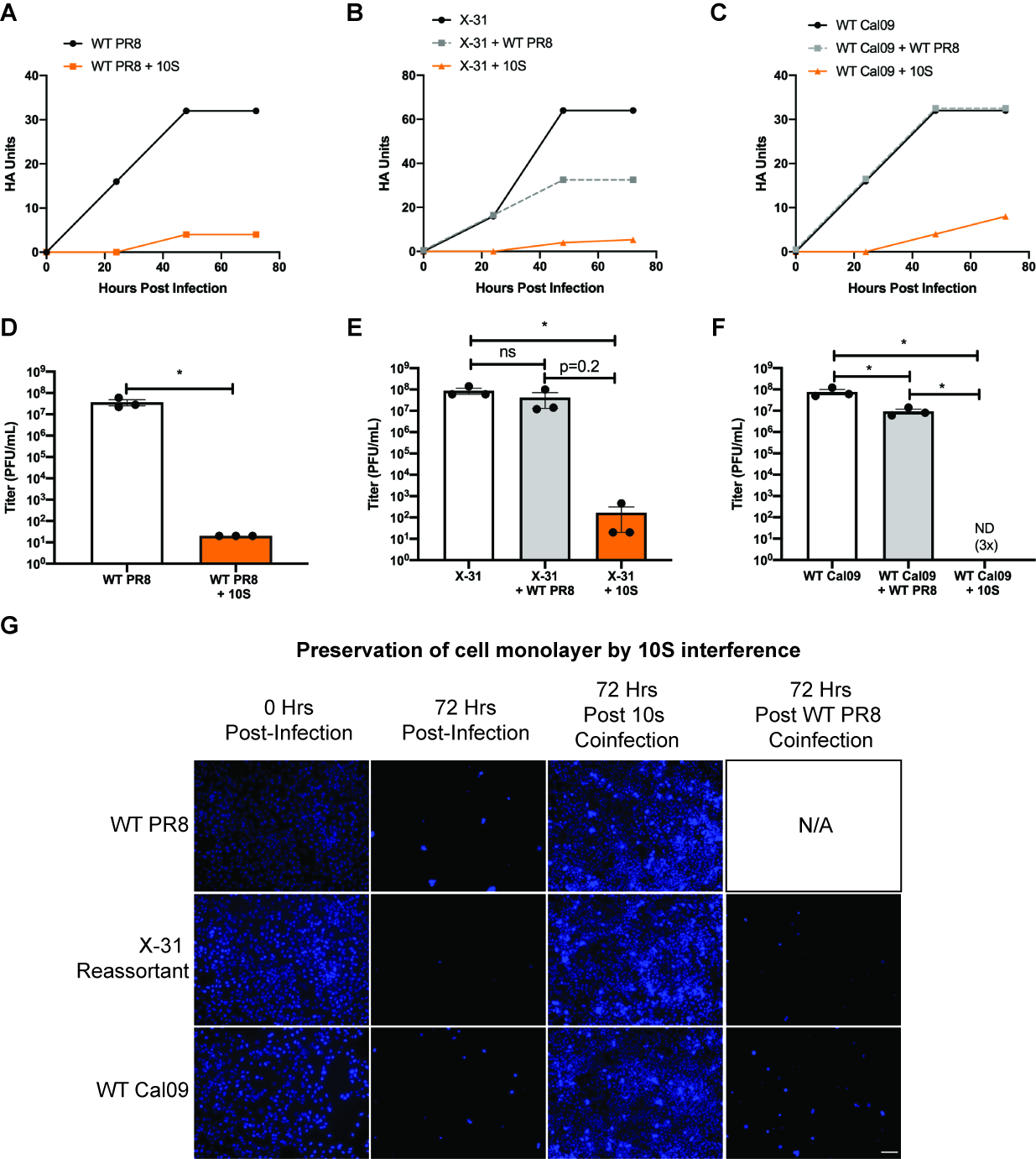

Supplement: S5 Fig — (A) HA units at 24, 48, and 72 hours post-infection of MDCK cells with either WT PR8 or coinfected with WT PR8 and the 10S virus. (B) HA units at 24, 48, and 72 hours post-infection of MDCK cells with either the X-31 reassortant or coinfected with X-31/WT PR8 or X-31/10S virus. (C) HA units at 24, 48, and 72 hours post-infection of MDCK cells with Cal09 or coinfected with Cal09/WT PR8 or Cal09/10S virus. (D) Endpoint titer 72 hours post-infection of MDCK cells with either WT PR8 or coinfected with WT PR8 and the 10S virus. (E) Endpoint titer 72 hours post-infection of MDCK cells with either the X-31 reassortant or coinfected with X-31/WT PR8 or X-31/10S virus. (F) Endpoint titer 72 hours post-infection of MDCK cells with Cal09 or coinfected with Cal09/WT PR8 or Cal09/10S virus. (G) Microscopy images of the cell monolayer before and after infection with either WT PR8, the H3N2 X-31 reassortant, and the pandemic H1N1 A/California/04/2009 virus as compared to after coinfection with either the 10S virus or WT PR8. For all graphs, error bars indicate the SEM, * represents a p-value of ≤ 0.05, and “ns” indicates that there was no significant difference. (TIF) [file ppat.1008098.s006.tif]
